# Reduced control of SARS-CoV-2 infection associates with lower mucosal antibody responses in pregnancy

**DOI:** 10.1128/msphere.00812-23

**Published:** 2024-03-01

**Authors:** Laura A. St Clair, Raghda E. Eldesouki, Jaiprasath Sachithanandham, Anna Yin, Amary Fall, C. Paul Morris, Julie M. Norton, Omar Abdullah, Santosh Dhakal, Caelan Barranta, Hana Golding, Susan J. Bersoff-Matcha, Catherine Pilgrim-Grayson, Leah Berhane, Andrea L. Cox, Irina Burd, Andrew Pekosz, Heba H. Mostafa, Eili Y. Klein, Sabra L. Klein

**Affiliations:** 1 W. Harry Feinstone Department of Molecular Microbiology and Immunology, Johns Hopkins University Bloomberg School of Public Health, Baltimore, Maryland, USA; 2 Department of Pathology, Division of Medical Microbiology, Johns Hopkins University School of Medicine, Baltimore, Maryland, USA; 3 Medical Genetics Unit, School of Medicine, Suez Canal University, Ismailia, Egypt; 4 National Institute of Allergy and Infectious Disease, National Institutes of Health, Bethesda, Maryland, USA; 5 Division of Viral Products, Center of Biologics Evaluation and Research, U.S. Food and Drug Administration, Silver Spring, Maryland, USA; 6 Office of Women’s Health, U.S. Food and Drug Administration, Silver Spring, Maryland, USA; 7 Division of Urology, Obstetrics, and Gynecology, Office of Rare Diseases, Pediatrics, Urologic and Reproductive Medicine and Office of New Drugs, Center for Drug Evaluation and Research, U.S. Food and Drug Administration, Silver Spring, Maryland, USA; 8 Department of Medicine, Johns Hopkins University School of Medicine, Baltimore, Maryland, USA; 9 Bloomberg Kimmel Institute for Cancer Immunotherapy, Johns Hopkins University School of Medicine, Baltimore, Maryland, USA; 10 Department of Obstetrics, Gynecology and Reproductive Sciences, University of Maryland School of Medicine, Baltimore, Maryland, USA; 11 Department of Emergency Medicine, Johns Hopkins University School of Medicine, Baltimore, Maryland, USA; 12 Center for Disease Dynamics, Economics, and Policy, United Nations Office for Disease Risk Reduction, Washington DC, USA; University of Michigan, Ann Arbor, Michigan, USA

**Keywords:** COVID-19, gestation, breakthrough infection, Omicron variant, Delta variant

## Abstract

Pregnant patients are at greater risk of hospitalization with severe COVID-19 than non-pregnant people. This was a retrospective observational cohort study of remnant clinical specimens from patients who visited acute care hospitals within the Johns Hopkins Health System in the Baltimore, MD–Washington DC, area between October 2020 and May 2022. Participants included confirmed severe acute respiratory syndrome coronavirus 2 (SARS-CoV-2)-infected pregnant people and matched non-pregnant people (the matching criteria included age, race/ethnicity, area deprivation index, insurance status, and vaccination status to ensure matched demographics). The primary dependent measures were clinical COVID-19 outcomes, infectious virus recovery, viral RNA levels, and mucosal anti-spike (S) IgG titers from upper respiratory tract samples. A total of 452 individuals (117 pregnant and 335 non-pregnant) were included in the study, with both vaccinated and unvaccinated individuals represented. Pregnant patients were at increased risk of hospitalization (odds ratio [OR] = 4.2; confidence interval [CI] = 2.0–8.6), intensive care unit admittance (OR = 4.5; CI = 1.2–14.2), and being placed on supplemental oxygen therapy (OR = 3.1; CI = 1.3–6.9). Individuals infected during their third trimester had higher mucosal anti-S IgG titers and lower viral RNA levels (*P* < 0.05) than those infected during their first or second trimesters. Pregnant individuals experiencing breakthrough infections due to the Omicron variant had reduced anti-S IgG compared to non-pregnant patients (*P* < 0.05). The observed increased severity of COVID-19 and reduced mucosal antibody responses particularly among pregnant participants infected with the Omicron variant suggest that maintaining high levels of SARS-CoV-2 immunity through booster vaccines may be important for the protection of this at-risk population.

**IMPORTANCE:**

In this retrospective observational cohort study, we analyzed remnant clinical samples from non-pregnant and pregnant individuals with confirmed severe acute respiratory syndrome coronavirus 2 (SARS-CoV-2) infections who visited the Johns Hopkins Hospital System between October 2020 and May 2022. Disease severity, including intensive care unit admission, was greater among pregnant than non-pregnant patients. Vaccination reduced recovery of infectious virus and viral RNA levels in non-pregnant patients, but not in pregnant patients. In pregnant patients, increased nasopharyngeal viral RNA levels and recovery of infectious virus were associated with reduced mucosal IgG antibody responses, especially among women in their first trimester of pregnancy or experiencing breakthrough infections from Omicron variants. Taken together, this study provides insights into how pregnant patients are at greater risk of severe COVID-19. The novelty of this study is that it focuses on the relationship between the mucosal antibody response and its association with virus load and disease outcomes in pregnant people, whereas previous studies have focused on serological immunity. Vaccination status, gestational age, and SARS-CoV-2 omicron variant impact mucosal antibody responses and recovery of infectious virus from pregnant patients.

## INTRODUCTION

Pregnant people are classified as at risk for severe COVID-19 complications (1–3). Analyses from the U.S. Centers for Disease Control and Prevention (CDC) show that among people with confirmed severe acute respiratory syndrome coronavirus 2 (SARS-CoV-2) infections from January 2020 to December 2021, pregnant people were five times more likely to be admitted to an intensive care unit (ICU), had a 76% greater risk of requiring invasive ventilation, and had a 3.3 times greater risk of death compared to non-pregnant people (4). Despite these increased risks, the immune responses to SARS-CoV-2 infection and the efficacy of SARS-CoV-2 vaccination in pregnant people remain understudied (5–8). Studies that have analyzed immune responses to SARS-CoV-2 infection and vaccination have largely focused on serological immunity, with limited analysis of the mucosal antibody response to SARS-CoV-2 infection (9) and its association with virus load, especially among pregnant people.

In this retrospective observational cohort study, remnant clinical specimens from pregnant and matched non-pregnant patients with confirmed positive SARS-CoV-2 infection who visited the Johns Hopkins Health System (JHHS) between October 2020 and May 2022 were analyzed for clinical outcomes, virus lineage, infectious virus recovery, viral RNA level, and assessment of mucosal anti-spike (S) IgG titers. Differences in each measure were compared between non-pregnant and pregnant people and stratified by vaccination status, trimester of pregnancy, and infecting SARS-CoV-2 variants.

## MATERIALS AND METHODS

### Subjects and sample selection

This was a retrospective observational cohort study that used remnant nasopharyngeal swabs (from symptomatic patients) or lateral mid-turbinate nasal swabs (from asymptomatic patients). At the time of sample collection, all patients visiting the JHHS, irrespective of the nature of their visit, were screened for SARS-CoV-2 infection. Clinical information of individuals was bulk-extracted from JHHS electronic medical records for those with a confirmed positive result following diagnostic screening. We excluded those who did not identify as female, whose sex at birth was recorded as male, or who chose not to disclose their sex at birth. After identifying samples from pregnant patients, propensity score matching was used to determine a cohort of control patients (3:1 ratio of control to pregnant patients). Psmatch2 in Stata was used to match the patients on age, vaccination status, race/ethnicity, area deprivation index (a measure of socioeconomic status), and insurance status using two methods. The first used no replacement (i.e., selection of best matches for every pregnant patient in the cohort), with a nearest neighbor of 4 with a caliper of 0.01, which was used to select additional patients that might be near close matches. This selection identified 117 pregnant individuals (84 unvaccinated and 33 vaccinated) and 335 demographically matched non-pregnant controls (244 unvaccinated and 91 vaccinated) for whom complete vaccination data, full sequencing data, and remnant clinical specimens were available for analysis (Table 1). In this study, vaccinated individuals were defined as those who either received two primary doses (Pfizer/BioNTech or Moderna mRNA-1273 vaccines) or received the primary doses and third booster dose prior to confirmed infection. Unvaccinated individuals were defined as individuals who had received no COVID-19 vaccine prior to infection. Individuals who were partially vaccinated were excluded from this study.

**TABLE 1:** Patients and samples used in this study^*a*^

Variable	Pregnant	First trimester	Second trimester	Third trimester	Not pregnant
*N*	117	28	36	53	335
Patient age					
Mean age	29.7	29.3	30.3	29.5	30.7
18–24, *n* (%)	26 (22.2%)	8 (28.6%)	7 (19.4%)	11 (20.8%)	72 (21.5%)
25–34, *n* (%)	64 (54.7%)	14 (50.0%)	18 (50.0%)	32 (60.4%)	157 (46.9%)
35–44, *n* (%)	27 (23.1%)	6 (21.4%)	11 (30.6%)	10 (18.9%)	106 (31.6%)
Race/ethnicity					
Black, *n* (%)	48 (41.0%)	14 (50.0%)	13 (36.1%)	21 (39.6%)	138 (41.2%)
Hispanic, *n* (%)	22 (18.8%)	2 (7.1%)	6 (16.7%)	14 (26.4%)	53 (15.8%)
Other, *n* (%)	13 (11.1%)	4 (14.3%)	3 (8.3%)	6 (11.3%)	30 (9.0%)
White, *n* (%)	34 (29.1%)	8 (28.6%)	14 (38.9%)	12 (22.6%)	114 (34.0%)
Ninth month, *n* (%)	26 (22.2%)	0 (0.0%)	0 (0.0%)	26 (49.1%)	0 (0.0%)
Area deprivation index	6.3	6.6	5.9	6.4	6.4
Charlson score	0	0	0	0	0
Vaccination status					
Unvaccinated, *n* (%)	84 (71.8%)	20 (71.4%)	27 (75.0%)	37 (69.8%)	244 (72.8%)
Vaccinated, *n* (%)	33 (28.2%)	8 (28.6%)	9 (25.0%)	16 (30.2%)	91 (27.2%)
Moderna mRNA-1273, *n* (%)	12 (10.2%)	2 (7.2%)	3 (8.3%)	7 (13.2%)	24 (7.2%)
Pfizer/BioNtech, *n* (%)	21 (18.0%)	6 (21.4%)	6 (16.7%)	9 (17.0%)	67 (20.0%)
Homologous booster, *n* (%)	7 (6.0%)	2 (7.1%)	2 (5.6%)	3 (5.7%)	46 (13.7%)
Heterologous booster, *n* (%)	1 (0.85%)	0 (0.0%)	0 (0.0%)	1 (1.9%)	3 (0.90%)

^*a*^ “Vaccinated” includes individuals who received a full two-dose mRNA vaccine regimen and/or received a booster dose prior to infection. “Unvaccinated” includes individuals who had not received any vaccine dose prior to infection. Partially vaccinated individuals were excluded from this study.

### Amplicon-based sequencing

The NEBNext ARTIC SARS-CoV-2 Companion Kit (VarSkip Short SARS-CoV-2 # E7660-L) was used for library preparation and sequencing using the Nanopore GridION. Base-calling of reads was conducted using the MinKNOW, followed by demultiplexing with guppybarcoder that requires barcodes at both ends. The Artic-ncov2019 medaka protocol was used for alignment and variant calling. Clades were determined using Nextclade beta v 1.13.2 (clades.nextstrain.org, last accessed 30 March 2022), and lineages were determined with Pangolin COVID-19 lineage Assigner (10). Sequences with coverage >90% and mean depth >100 were submitted to the GISAID database and are available at the Johns Hopkins Research Data Repository (https://doi.org/10.7281/T1/IRMGH8).

### SARS-CoV-2 PCR

After clinical diagnosis, samples were retested using the CDC-designed primers and probes for the N gene to assess viral RNA levels (cycle threshold or Ct) (11). An equivalent distribution of data between samples collected from NP swabs and lateral mid-turbinate nasal swabs was observed; as such, analysis of Ct values did not control for the sample type.

### Infectious SARS-CoV-2 recovery

TMPRSS2 VeroE6 cells (RRID: CVCL_YQ49) obtained from the cell repository of the National Institute of Infectious Diseases (12) were cultured at 37°C/5% CO_2_ using culture media (CM) consisting of Dulbecco’s modified Eagle medium (Sigma Aldrich), supplemented with 2 mM L-glutamine (Invitrogen), 100 µg/mL penicillin/streptomycin (Invitrogen), and 10% filter-sterilized fetal bovine serum (Gibco). For virus isolation, cells plated in 24-well dishes had the culture media replaced with 350 µL of infection media (CM with fetal bovine serum reduced to 2.5%), followed by the addition of 150 µL of swab specimen. After incubation for 2 hours at 37°C, the inoculum was removed and replaced with 500 µL infection media. The cells were monitored daily for the appearance of SARS-CoV-2 cytopathic effect (CPE), and the presence of SARS-CoV-2 genomes in CPE-positive samples was confirmed by reverse transcriptase PCR.

### Indirect enzyme-linked immunosorbent assays

This protocol was adapted from published protocols (5, 13) and was modified to assess virus-specific IgG from viral transport media (VTM). Ninety-six-well plates were coated with full-length ancestral S protein (SeroNet) and incubated overnight at 4°C. Following plate coating, all remaining blocking and incubation steps occurred at room temperature and in the dark. Plates were washed and blocked for 1 hour. Prior to use, samples were heat-inactivated at 56°C for 1 hour, then prepared in twofold serial dilutions (1:4–1:512). Negative controls using pooled VTM from COVID-19-negative patients were plated at a final concentration of 1:4. A monoclonal antibody against SARS-CoV-2 S protein (Sino Biological, 40150-D001) was plated at a final concentration of 1:5,000 for a positive control. The blocking solution was removed, and samples were added to the plates and incubated for 2 hours. Plates were washed three times, 50 µL of secondary antibody (1:5,000 dilution of HRP-conjugated goat anti-human IgG; Invitrogen #A18823) was added to each well, and plates were incubated for 1 hour. Plates were washed, and all residual liquid was removed. SIGMAFAST o-phenylenediamine dihydrochloride solution (Millipore Sigma) was added to each well, and plates were incubated for 10 minutes. Three-molar HCl was added to each well to stop the reaction, and the optical density of each plate was read at 490 nm using a SpectraMax i3 ELISA Plate Reader (BioTek Instruments). The cutoff values were calculated by adding the average of all negative control OD values and three times the standard deviation of the negative control values. Values were considered positive (responders) if at or above the cutoff value and negative (non-responders) if below the cutoff. All samples were processed as a single batch together, mitigating potential batch effects on the cutoff and OD values.

### Statistical analyses

Comparisons of clinical characteristics, infectious virus recovery, and between anti-S IgG responders and non-responders were tested using two-sided Fisher’s exact test with a Baptista–Pike odds ratio (in cases with non-zero values) or Woolf logit odds ratio (in cases with zero values). For anti-S IgG, the area under the curve (AUC) values were calculated by plotting the normalized optical density values against the sample dilution in order to obtain the total peak area from the OD values as described in reference 14. A two-way ANOVA using Tukey’s multiple comparisons was used to assess differences in anti-S IgG AUC among groups, as well as differences in SARS-CoV-2 N Ct values among groups. Regression models (logistic and linear) were used to investigate the association of immunological measures (CPE, viral RNA level, and anti-spike IgG) with pregnancy and vaccination. An interaction term of the predictor variables was also included in the statistical models to allow for the predicted probabilities to vary by pregnancy and vaccination status. All analyses were performed using either Prism software version 9.5 (GraphPad) or Stata version 17.0 (StataCorp).

## RESULTS

### Clinical outcomes from COVID-19 among pregnant and non-pregnant patients

The clinical outcomes between pregnant and non-pregnant patients with confirmed SARS-CoV-2 infections differed. While pregnant patients were less likely to report symptoms than non-pregnant patients (OR = 0.41; CI = 0.23–0.71; *P* = 0.003), among symptomatic individuals, pregnant patients were more likely to require hospitalization (OR = 4.2; CI = 2.0–8.6, *P* = 0.0003) or be admitted to the ICU (OR = 4.5; CI = 1.2–14.2, *P* = 0.02) with COVID-19 as their primary reason for admission (OR = 3.1; CI = 1.4–6.8; *P* = 0.009) (Table 2). In addition, pregnant patients were more likely to be placed on supplemental oxygen therapy than non-pregnant patients (OR = 3.1; CI = 1.3–6.9, *P* = 0.012) (Table 2). When stratified by vaccination status, vaccination reduced the proportion of pregnant patients requiring hospitalization and supplemental oxygen therapy (Table 2).

**TABLE 2:** Differences in COVID-19 severity between non-pregnant and pregnant patients

Variable	Pregnant	First trimester	Second trimester	Third trimester	Non-pregnant	Odds pregnant vs non-pregnant total (CI)	*P*-value
*N*	117	28	36	53	335		
Symptomatic total, *n* (%)	91 (77.7%)	25 (89.3%)	32 (88.9%)	34 (64.1%)	300 (89.6%)	0.41 (0.23–0.71)	0.003
Unvaccinated, *n* (%)	63 (69.2%)	18 (72.0%)	24 (75.0%)	21 (61.8%)	220 (73.3%)	0.33 (0.17–0.61)	0.0014
Vaccinated, *n* (%)	28 (30.8%)	7 (28.0%)	8 (25.0%)	13 (38.2%)	80 (26.7%)	0.77 (0.26–2.1)	0.76
Hospitalization total, *n* (%)	17 (14.5%)	0 (0.0%)	3 (8.3%)	14 (26.4%)	13 (3.9%)	4.2 (2.0–8.6)	0.0003
Unvaccinated, *n* (%)	12 (70.6%)	0 (0.0%)	2 (16.7%)	10 (83.3%)	13 (100%)	5.9 (2.6–12.8)	<0.0001
Vaccinated, *n* (%)	5 (29.4%)	0 (0.0%)	1 (20.0%)	4 (80.0%)	0 (0.0%)	37.7 (2.0–706.6)	0.0009
COVID reason for admission total, *n* (%)	13 (11.1%)	0 (0.0%)	2 (5.6%)	11 (20.7%)	13 (3.9%)	3.1 (1.4–6.8)	0.009
Unvaccinated, *n* (%)	10 (76.9%)	0 (0.0%)	2 (100%)	8 (72.7%)	13 (100%)	3.0 (1.2–7.4)	0.017
Vaccinated, *n* (%)	3 (23.1%)	0 (0.0%)	0 (0.0%)	3 (27.2%)	0 (0.0%)	24.0 (1.2–481.0)	0.013
ICU admittance total, *n* (%)	6 (5.1%)	0 (0.0%)	1 (2.8%)	5 (9.4%)	4 (1.2%)	4.5 (1.2–14.2)	0.02
Unvaccinated, *n* (%)	3 (50.0%)	0 (0.0%)	0 (0.0%)	3 (60.0%)	3 (75.0%)	3.6 (0.8–15.6)	0.13
Vaccinated, *n* (%)	3 (50.0%)	0 (0.0%)	1 (100%)	2 (40.0%)	1 (25.0%)	26.3 (3.7–341.9)	0.005
Supplemental O_2_, *n* (%)	11 (9.4%)	0 (0.0%)	2 (5.6%)	9 (17.0%)	11 (3.3%)	3.1 (1.3–6.9)	0.01
Unvaccinated, *n* (%)	7 (63.6%)	0 (0.0%)	2 (100.0%)	5 (55.6%)	11 (100.0%)	2.4 (9.2–6.4)	
Vaccinated, *n* (%)	4 (36.4%)	0 (0.0%)	0 (0.0%)	4 (44.4%)	0 (0.0%)	29.6 (1.5–568.7)	0.004

### Distribution of SARS-CoV-2 variants among pregnant and non-pregnant patients

Whole genome sequencing (WGS) results were used to classify infecting SARS-CoV-2 variants into one of five categories: ancestral lineages (i.e., those circulating prior to Alpha), Alpha variant, Delta variant, Omicron variant (through BA.2.12.1), and other. Among unvaccinated individuals, most samples were from infections prior to vaccine availability and were predominately caused by ancestral lineages (40% in non-pregnant people and 32% in pregnant people); samples from infections by all other variants, however, were proportionally represented (Table 3). As emergency use authorization of both the Pfizer/BioNTech and Moderna mRNA-1273 vaccines coincided with the emergence and dominance of the Alpha variant, many samples collected from the vaccinated non-pregnant and pregnant cohort were individuals experiencing breakthrough infections from either the Delta variant (53% and 24%, respectively) or Omicron variants (38% and 73%, respectively) (Table 3).

**TABLE 3:** SARS-CoV-2 lineage distribution

Variable	Ancestral	Alpha	Delta	Omicron	Other	Total
Unvaccinated						
Non-pregnant, *n* (%)	97 (40%)	58 (24%)	32 (13%)	23 (9%)	34 (14%)	244
Pregnant, *n* (%)	27 (32%)	12 (14%)	14 (17%)	23 (27%)	8 (10%)	84
Vaccinated						
Non-pregnant, *n* (%)	5 (6%)	1 (1%)	48 (53%)	35 (38%)	2 (2%)	91
Pregnant, *n* (%)	0 (%)	0 (0%)	8 (24%)	24 (73%)	1 (3%)	33

### SARS-CoV-2 virus RNA level and recovery of infectious virus from upper respiratory samples

**Fig 1:**
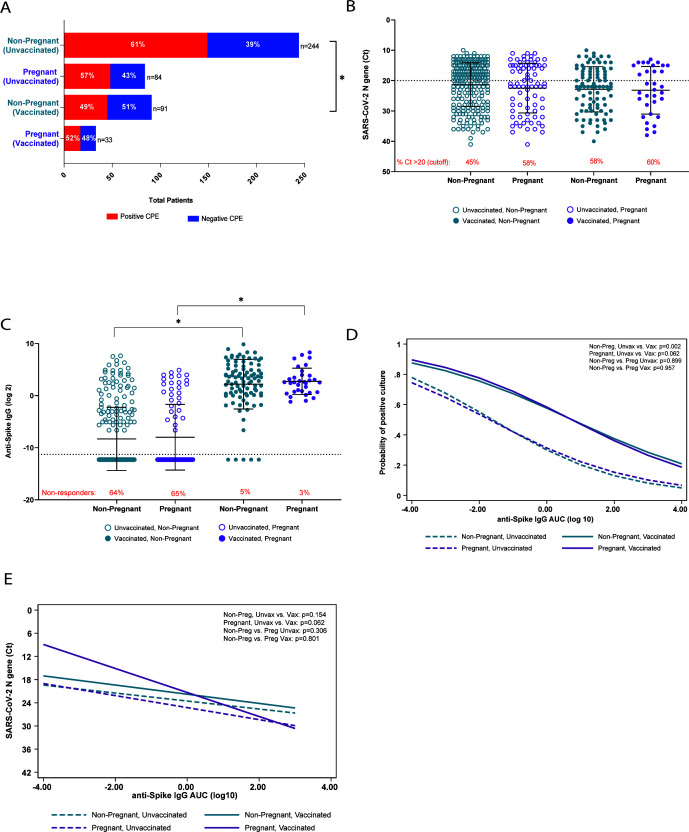
SARS-CoV-2 viral RNA levels and antibody responses stratified by pregnancy and vaccination status. Remnant clinical upper respiratory tract specimens were used to determine rates of infectious virus recovery (**A**), viral RNA level (**B**), and anti-S (ancestral) IgG titers calculated as AUC (**C**) from mucosal swab samples. In panel **A**, a positive CPE in tissue culture was indicative of the presence of infectious virus. The dashed line in panel **B** represents the cutoff value (Ct ≤ 20) between high viral RNA and low viral levels, and the red text indicates the percentage of participants with Ct values > 20 (low viral RNA levels). The dashed line in panel **C** represents the limit of detection, and the red text indicates the percentage of non-responders (results below the limit of detection). Multivariable logistic regression was used to analyze the association between anti-spike IgG AUC and infectious virus-positive cultures (**D**) or viral RNA levels (**E**). Variables were continuous, and comparisons by vaccination and pregnancy status are shown. Analysis included Fisher’s exact test (**A**), two-way ANOVAs with Tukey’s multiple comparisons test (**B and C**), and multivariable logistic regression (**E**). ^*^P < 0.05. anti-S IgG, anti-ancestral strain spike immunoglobulin G.

To evaluate if the differences in clinical severity between non-pregnant and pregnant patients were due to differences in virus load, we compared infectious virus recovery and viral RNA levels (Ct values) for each group. Because there were no statistical differences in the days to symptom onset between symptomatic non-pregnant (2.2 ± 2.6 days) and pregnant (2.4 ± 3.4 days) people within this cohort, these analyses were conducted regardless of the days to symptom onset and whether the patient was symptomatic or asymptomatic at the time of collection, consistent with our previously published studies (15). The number of samples from which infectious virus was recovered was significantly lower among non-pregnant vaccinated than unvaccinated patients (*P* < 0.05; Fig. 1A). While a similar trend was noted between unvaccinated and vaccinated pregnant patients, this did not reach statistical significance. There were no statistical differences in the rates of infectious virus recovery between non-pregnant and pregnant patients, regardless of vaccination status. Viral RNA levels were similarly distributed between pregnant and non-pregnant patients, with no statistical differences observed (Fig. 1B). Additionally, we assessed whether there were differences between the number of individuals with high (Ct > 20; low viral RNA levels) versus low (Ct ≤ 20; high viral RNA levels) viral RNA levels within pregnant and non-pregnant patients. While greater percentages of vaccinated non-pregnant and pregnant people had lower viral levels (58% and 60%, respectively) than their unvaccinated counterparts (45% and 58%, respectively), these differences were not statistically significant (Fig. 1B, red text).

### Comparisons of mucosal anti-S IgG titers between pregnant and non-pregnant patients

Although previous reports suggest that pregnant patients have reduced antibody responses to SARS-CoV-2 infection (5, 16–18), these studies focused solely on serum antibody responses. As SARS-CoV-2 infection initiates in the upper respiratory tract, we sought to evaluate whether differences in mucosal IgG responses between non-pregnant and pregnant people may account for differences in clinical severity. IgG was quantified rather than IgA because titers of IgG and seroconversion rates of IgG are higher in response to mRNA vaccines (17, 19–22). Vaccinated individuals had greater anti-S IgG titers than unvaccinated individuals, regardless of pregnancy status (*P* < 0.0001; Fig. 1C). The average time between completion of vaccination and infection was similar between non-pregnant (176 ± 85 days) and pregnant (187 ± 95 days) patients. Proportions of individuals with undetectable anti-S IgG (i.e., non-responders) were greater in unvaccinated people compared to vaccinated people (non-pregnant: *P* < 0.0001; pregnant: *P* < 0.0001), but there were no statistically significant differences between pregnant and non-pregnant people within vaccination groups (Fig. 1C, red text). The correlation between mucosal anti-S IgG titers and infectious virus recovery and between anti-S IgG titers and viral RNA Ct values was examined as a proxy to assess whether there were differences in the antiviral activity of antibodies produced by non-pregnant and pregnant patients. In these regression models, there was a strong inverse association between anti-S IgG AUC and the probability of recovering infectious virus (Fig. 1D) as well as viral RNA levels (Fig. 1E) among all participants, regardless of vaccination or pregnancy status. Notably, when the variables for time post-symptom onset (excluding asymptomatic individuals; non-pregnant, *N* = 35; pregnant, *N* = 26), time from completion of vaccination to infection, or infecting variant were included in the regression models, the association between anti-S IgG AUC and the probability of recovering infectious virus as well as between anti-S IgG and viral RNA Ct values remained unchanged (data not shown).

### Trimester of pregnancy influences mucosal immunity in pregnant, vaccinated patients

**Fig 2:**
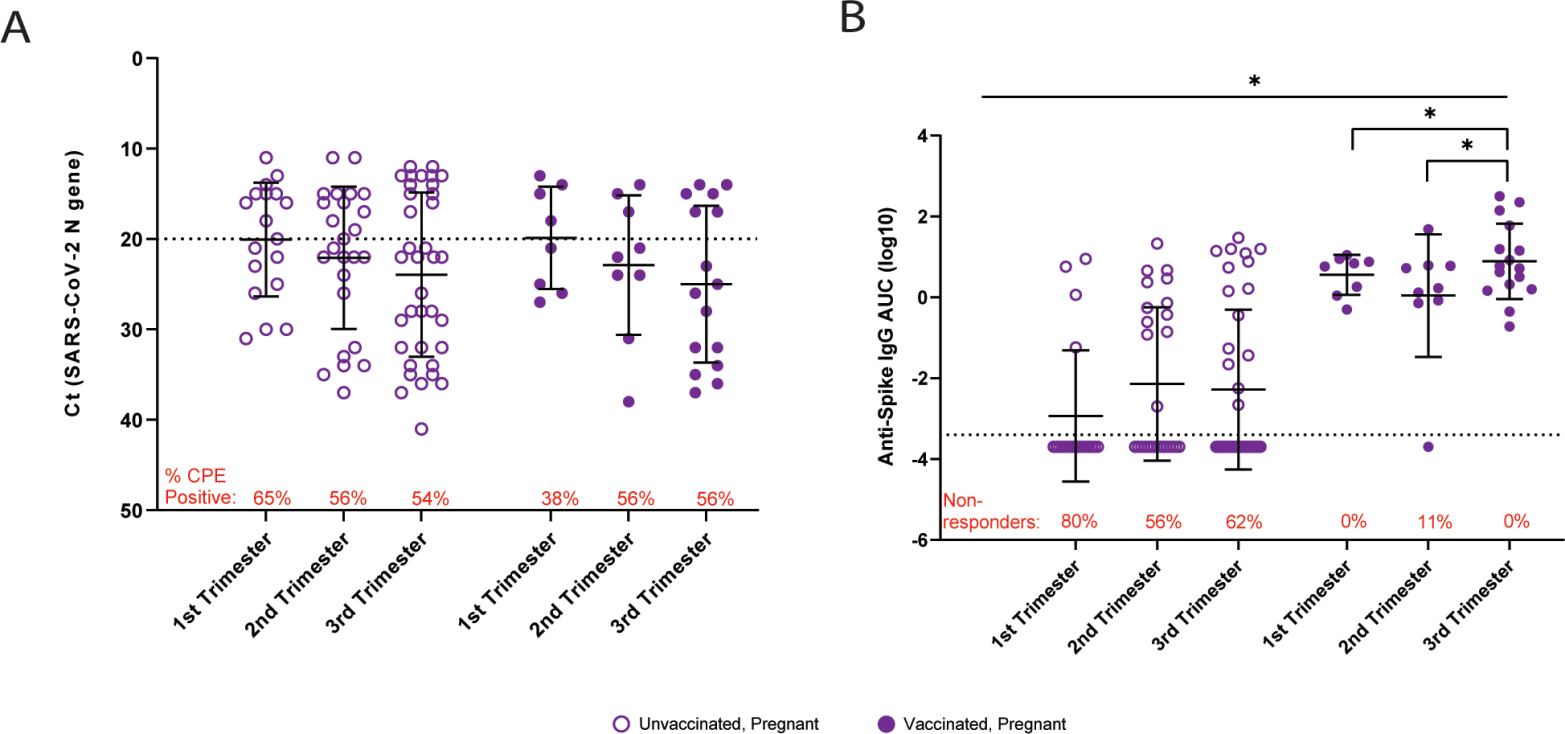
The effects of gestational age on mucosal viral RNA levels and antibody responses. Study participants were divided into unvaccinated and vaccinated pregnant patients according to the trimester of pregnancy and analyzed to assess differences in viral RNA levels (**A**) and anti-S IgG AUC (**B**). The red text indicates the percentage of individuals with recoverable infectious virus (**A**) or the percentage of IgG non-responders (i.e., those with anti-S IgG AUC below the limit of detection; **B**). Two-way ANOVA with Tukey’s multiple comparisons test. ^*^P < 0.05.

Other studies published through the Delta wave (4, 23, 24) and during the Omicron wave highlight that pregnancy is a risk factor for more severe COVID-19 outcomes, with outcomes being worse during the third trimester of pregnancy (18, 25). We examined the relationship between gestational age, viral RNA level, mucosal anti-S IgG AUC values, and recovery of infectious virus, regardless of days to symptom onset or whether the patients were symptomatic or asymptomatic. Although no statistical differences in viral RNA level (Fig. 2A) or recovery of infectious virus (Fig. 2A, red text) were observed across trimesters of pregnancy, a trend of reduced viral RNA across trimesters was observed, with the lowest values being recorded in the third trimester for both unvaccinated and vaccinated pregnant patients. Among vaccinated pregnant people, anti-S IgG AUC values were greater in the third trimester compared to either the first (*P* < 0.05) or second (*P* < 0.05) trimesters of pregnancy (Fig. 2B). Proportions of non-responders (i.e., those with undetectable anti-S IgG) within each trimester were greater in unvaccinated compared to vaccinated pregnant patients (first trimester: *P* = 0.0002; second trimester: *P* = 0.02; third trimester: *P* = 0.002) and were not statistically different between trimesters within vaccination groups (Fig. 2B, red text). The severity of COVID-19 was positively associated with greater serum antibody responses in other cohort studies (26, 27). Our data suggest that mucosal antibody responses to SARS-CoV-2 infection are greater later in pregnancy, when more severe disease is observed.

### Pregnant patients infected with Omicron variants have reduced mucosal anti-S IgG levels

**Fig 3:**
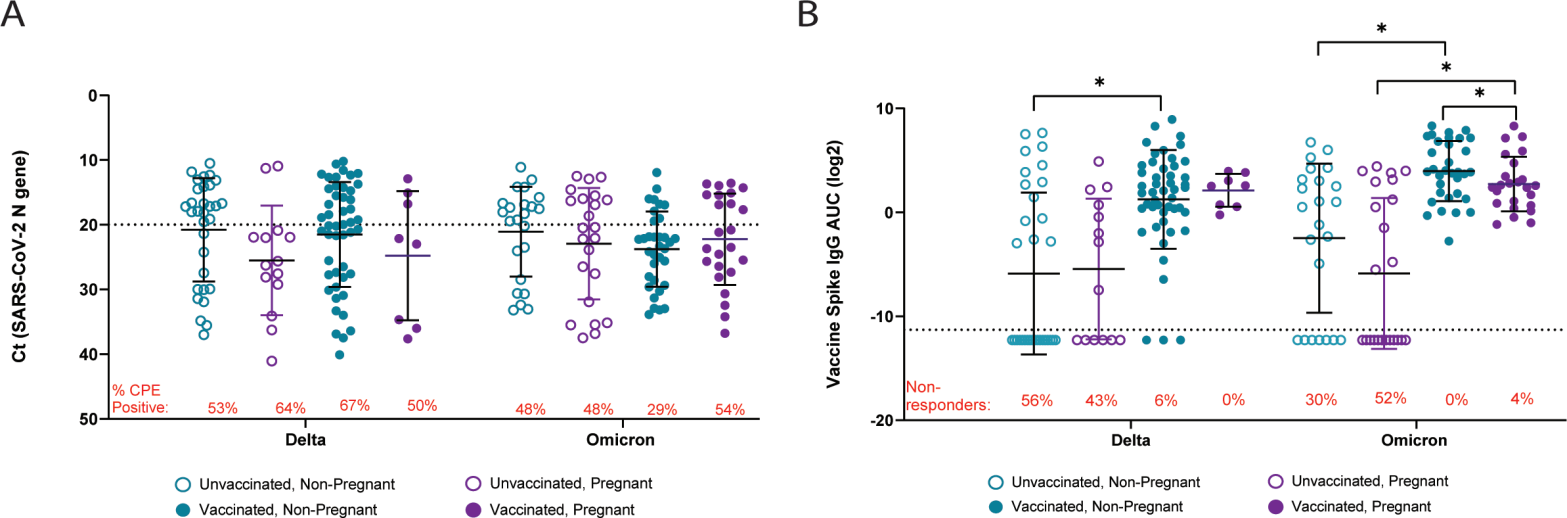
Analysis of mucosal viral RNA levels and antibody responses to Delta and Omicron breakthrough infections during pregnancy. Samples were classified according to infecting strain (Delta or Omicron), pregnancy status, and vaccination status and reanalyzed to assess differences in viral RNA level (**A**) and anti-S (ancestral/vaccine strain) IgG AUC (**B**). Two-way ANOVA with Tukey’s multiple comparisons tests (**A and B**). ^*^P < 0.05.

This patient cohort included individuals infected with both Delta and Omicron (through BA.2.12.1) variants. We conducted an additional analysis of pregnancy-associated differences based on the infecting variant. No differences in viral RNA level were detected among either pregnant or non-pregnant patients (Fig. 3A). Pregnant, vaccinated individuals infected with Omicron, but not Delta, variants had significantly lower mucosal anti-S IgG AUC values than non-pregnant, vaccinated patients (*P* < 0.05; Fig. 3B). In contrast, anti-S IgG AUC values were comparable between unvaccinated pregnant and non-pregnant patients infected with either Delta or Omicron variants. The proportion (Fig. 3B, red text) of unvaccinated, non-pregnant patients with non-detectable anti-S IgG titers was lower among those infected with Omicron variants compared to Delta (*P* = 0.01) but was higher among unvaccinated pregnant patients (*P* = 0.0003). Similar observations were made among vaccinated individuals but were not statistically significant.

## DISCUSSION

The COVID-19 pandemic raised awareness about pregnant people being at greater risk for severe complications arising from viral infection (1, 4, 28). Existing serological evidence in SARS-CoV-2 infection demonstrates that pregnant patients have enhanced inflammatory responses and reduced humoral responses compared to non-pregnant patients (5, 6, 29, 30). In a retrospective cohort of pregnant and non-pregnant patients with confirmed SARS-CoV-2 infection, we observed that disease severity, including ICU admission and oxygen supplementation, was greater among pregnant than non-pregnant patients. We further explored the role of vaccination in mucosal immunity and recovery of live SARS-CoV-2 and viral RNA from the upper respiratory tract. Vaccination reduced recovery of infectious virus in non-pregnant, but we did not observe the same effect on pregnant patients, suggesting that vaccine-induced immunity and protection might be reduced during pregnancy, as previously reported for other infectious diseases (1). These findings may provide mechanistic insights into how pregnant people are at greater risk of severe COVID-19, including breakthrough infections with variants of concern following receipt of the monovalent COVID-19 vaccines.

Among pregnant patients with confirmed SARS-CoV-2 infection, reduced mucosal antibody responses were associated with greater infectious virus recovery and viral RNA levels, especially among patients infected with the Omicron variant, which is consistent with other studies (31, 32). These data highlight the value of updating COVID-19 vaccine platforms annually to protect pregnant people against novel variants as cross-protection at the mucosal site of infection is reduced by pregnancy. Pregnant people were not included in phase III clinical trials for any of the vaccine candidates or boosters (33), and limited data are available from people who became pregnant while participating in vaccine trials (34, 35). Additional studies evaluating vaccine efficacy and the use of SARS-CoV-2 therapeutic agents (e.g., monoclonal antibodies) are necessary to ensure that the same correlates of protection apply to this high-risk population (36).

The primary limitation of this study is the small sample size. While some comparisons were able to reach statistical significance with the limited sample sizes, we were unable to give adequate statistical consideration for additional comparisons or potential confounding variables (e.g., time since symptom onset and time between vaccination and sample collection). This was due both to incomplete charting data (e.g., 77 symptomatic participants without a reported date of symptom onset) and to the use of convenience samples, which limited our ability to control for time between vaccination and sample collection. Our observations need to be verified in a larger clinical cohort. Moreover, only upper respiratory samples were collected, and no serum samples were available for additional analyses (e.g., virus neutralization or cross-reactivity with spike proteins from variants of concern). For clinical outcomes, pregnant patients in our study were reportedly less symptomatic than non-pregnant people; this was, however, based on self-reporting from a general list of questions that may not distinguish COVID-19-related illness from pregnancy-associated symptoms (e.g., fatigue, muscles or body aches, headache, digestive issues, nausea, or vomiting). Symptomatic COVID-19 cases among pregnant patients may not be accurately represented.

## Data Availability

Data will be made available upon request. Sequences with coverage >90% and mean depth >100 were submitted to the GISAID database and and are available at the Johns Hopkins Research Data Repository (https://doi.org/10.7281/T1/IRMGH8).
